# NETWORKED 3B: a novel protein in the actin cytoskeleton-endoplasmic reticulum interaction

**DOI:** 10.1093/jxb/erx047

**Published:** 2017-03-28

**Authors:** Pengwei Wang, Patrick J Hussey

**Affiliations:** 1 Department of Biosciences, Durham University, South road, Durham, DH1 3LE,UK; 2 College of Horticulture and Forestry Sciences, Huazhong Agricultural University, Wuhan 430070, Hubei Province, PR China

**Keywords:** Actin cytoskeleton, endoplasmic reticulum, NET superfamily, endomembrane system, *N. benthamiana*.

## Abstract

In plants movement of the endoplasmic reticulum (ER) is dependent on the actin cytoskeleton. However little is known about proteins that link the ER membrane and the actin cytoskeleton. Here we identified a novel protein, NETWORKED 3B (NET3B), which is associated with the ER and actin cytoskeleton *in vivo*. NET3B belongs to a superfamily of plant specific actin binding proteins, the NETWORKED family. NET3B associates with the actin cytoskeleton *in vivo* through an N-terminal NET actin binding (NAB) domain, which has been well-characterized in other members of the NET family. A three amino acid insertion, Val-Glu-Asp, in the NAB domain of NET3B appears to lower its ability to localize to the actin cytoskeleton compared with NET1A, the founding member of the NET family. The C-terminal domain of NET3B links the protein to the ER. Overexpression of NET3B enhanced the association between the ER and the actin cytoskeleton, and the extent of this association was dependent on the amount of NET3B available. Another effect of NET3B overexpression was a reduction in ER membrane diffusion. In conclusion, our results revealed that NET3B modulates ER and actin cytoskeleton interactions in higher plants.

## Highlight

Expressing high levels of GFP-tagged NETWORKED 3B (NET 3B) in plant cells enhances the association between the endoplasmic reticulum and actin cytoskeleton.

## Introduction

Remodelling of the endoplasmic reticulum (ER) in plants is mainly dependent on the actin cytoskeleton (Boevink *et al.*, 1998; Sparkes *et al.*, 2009). Recent studies have also suggested a role for microtubules in slow movement of the ER (Hamada *et al.*, 2014). Although several studies have indicated that certain actin regulatory proteins, such as Scar/Wave complexes and capping proteins, are localizelocalized to the ER membrane (Zhang *et al.*, 2013; Jimenez-Lopez *et al.*, 2014; Wang *et al.*, 2016a), only members of the myosin XI family have been shown to modulate the ER network so far (Sparkes *et al.*, 2009; Yokota *et al.*, 2011). ER dynamics, organelle movement, actin organization and plant development are affected by knocking out myosin expression or by overexpressing myosin dominant negative constructs (Sparkes *et al.*, 2008; Peremyslov *et al.*, 2010; Griffing *et al.*, 2014). Several independent studies have demonstrated that myosin XI-K is enriched in the ER fraction (Ueda *et al.*, 2010) and localized to motile membrane puncta (Peremyslov *et al.*, 2012).

The ER can be divided into two morphological classes: tubular ER and cisternal ER. The transition between these two types of ER membrane is poorly understood. One major structural component of the ER is the reticulon protein family, a family of integral membrane proteins that are able to induce the formation of ER tubules (Tolley *et al.*, 2008; Sparkes *et al.*, 2010). They consist of four transmembrane domains organized into a W shape. They predominantly localize to regions of the ER membrane where there is high membrane curvature (Knox *et al.*, 2015; Kriechbaumer *et al.*, 2015; Breeze *et al.*, 2016). The tubular structure of the ER is also maintained by a dynamin-like GTPase, atlastin, which is required for network formation and homotypic ER membrane fusion (Hu *et al.*, 2009). The plant homologue of atlastin is root hair defective 3 (RHD3) and there are several similar proteins called RHD3-like proteins (Zheng *et al.*, 2004; Chen *et al.*, 2011). RHD3 interacts with reticulons and expression of dominant negative constructs affects ER morphology and Golgi movement (Lee *et al.*, 2013).

In addition, the structure of cortical ER is also maintained by ER-plasma membrane contact sites (EPCS) (Wang *et al.*, 2016*b*). Depletion of ER-plasma membrane tethering proteins, such as Scs2, extended synaptotagmins or Ist2, affects the formation of EPCS and the structure of the cortical ER network (Fernández-Busnadiego *et al.*, 2015; Kralt *et al.*, 2015; Siao *et al.*, 2016). In plants, EPCS integrate the actin and microtubule networks with a complex of various proteins (Wang *et al.*, 2014). This protein complex includes VAP27, which is a homologue of Scs2 (Wang *et al.*, 2016c) and NET3C. Both localizelocalize to the ER membrane and plasma membrane. NET3C belongs to a plant specific actin binding protein superfamily, the NET family (Deeks *et al.*, 2012). NET3C binds to the actin cytoskeleton through its N-terminal NET actin binding (NAB) domain and is recruited to the membrane by its C-terminal sequence (Wang *et al.*, 2014). This special feature suggests that NET proteins act as adapters that link membranes to the actin cytoskeleton (Wang and Hussey, 2015).

In this study, we demonstrate that NET3B localizes to the actin cytoskeleton and the ER membrane *in vivo*, where it acts as an adapter between these two structures. We show that the NAB domain of NET3B is unique amongst the NET superfamily due to the insertion of three amino acids, Val-Glu-Asp (Hawkins *et al.*, 2014), which affects its ability to co-localize with the actin cytoskeleton *in vivo*. The C-terminal domain of NET3B is responsible for its interaction with the ER. Overexpressing NET3B increases the association of the ER with the actin cytoskeleton. This supports the notion that NET3B acts as a linker between the ER and actin cytoskeleton. However, T-DNA mutants of NET3B, *net2b-1* and *net2b-2*, show no significant morphological phenotype. Finally, we propose that NET3B mediates the ER and actin cytoskeleton interaction *in vivo* - a mechanism that is so far specific to higher plants.

## Materials and methods

### Molecular biology

The primers used in the vector constructions are listed in Supplementary Table S1 available at *JXB* online. NET3B (At4g03153) full length cDNA was amplified from CDS cDNA template (TAIR) with gene specific primers. The domain deletion mutants of NET3B were generated by overlapping PCR with the appropriate primers. Fluorescent protein fusions of NET3B, either full length or deletion mutants, were generated using Gateway recombination (Invitrogen) into GFP/RFP destination vectors derived from pMDC83. The NET3B promoter::GUS construct was made by fusing 2 kb of sequence upstream of the coding sequence of NET3B to the GUS reporter coding sequence (Supplementary Table S2).

### Plant growth and transformation

The transformation and growth of *Arabidopsis thaliana* and *Nicotiana benthamiana* were performed as described by Wang *et al.* (2014). The two NET3B SALK T-DNA insertion lines were ordered from the Nottingham Arabidopsis Stock Centre. Homozygosity was confirmed by PCR using gene specific primers and a T-DNA specific primer. Arabidopsis wild type (col-0) and *net3b-2* lines were transformed with RFP-HDEL by floral dipping. Tissues from Arabidopsis lines expressing NET3B::GUS were incubated in GUS staining solution [100 mM phosphate buffer, 10 mM EDTA, 0.1% (v/v) Triton X-100, 5 mM potassium ferricyanide, 5 mM potassium ferrocyanide, 1mM X-Gluc (5-bromo-4-chloro-3-indolyl-β-D-glucuronide)] for three hours at 37 °C. Before imaging, samples were decoloured by washing with 70% ethanol overnight.

### Confocal microscopy

Live cell imaging was carried out using a Leica SP5 laser scanning confocal microscope with a 63x oil immersion lens. For the GFP/YFP combination, GFP was excited at 458 nm and detected at 470–510 nm, while YFP was excited at 514 nm and detected at 550–580 nm. CFP/GFP/RFP combinations were excited at 405 nm, 488 nm and 543 nm and detected at 450–490 nm, 510–550 nm and 590–650 nm, respectively. Fluorescence recovery after photbleaching (FRAP) experiments were performed as described by Wang *et al.* (2011). All images presented here are representative of at least three independent experiments.

### Immunofluorescence

In order to make a polyclonal antibody against NET3B, DNA corresponding to amino acid residues 157–215 of NET3B was cloned into pGAT4 plasmid. This resulted in the incorporation of an N-terminal His tag into the expressed protein. The recombinant protein was generated in *Escherichia coli* (Rosseta 2, Novagen) and purified using nickel agarose beads (Qiagen). Polyclonal antibodies were raised in mice as described by Deeks *et al.* (2012). The specificity of the antiserum was tested on a western blot using total protein extract from two-week-old Arabidopsis seedlings. Immunofluorescence with freeze shattering was performed as described by Zhang *et al.* (2013). Antibodies were diluted and used at 1:100 for NET3B and 1:500 for BIP2 (Agrisera), followed by secondary antibody incubation with TRITC-conjugated anti-mouse IgG and FITC-conjugated anti-rabbit IgG (Jackson ImmunoResearch).

### High-speed centrifugation and microsomal isolation

*N. benthamiana* leaves expressing NET3B-GFP were used for total microsomal fraction isolation. Approximately 0.1 g of leaf tissue was homogenized in 12% (w/v) sucrose buffer containing 50 mM Tris hydrochloride at pH 7.6), 100 mM sodium chloride and 5 mM EDTA. Ultracentrifugation was performed at 55000 rpm using a Beckman TLA-100 rotor for 60 min. Both total microsome pellet and supernatant were mixed with SDS buffer, fractionated by SDS-PAGE and subsequently subjected to western blotting. For immunoblotting, primary antibodies of anti-GFP (Abcam), anti-BIP2 (Agrisera) and anti-actin (C4, Millipore) were used at 1:1000, 1:1000 and 1:500, respectively. Horseradish peroxidase-conjugated secondary antibody and ECL reagent (GE Heathcare) were used for developing the membrane.

## Results and discussion

### NET3B links the ER membrane and the actin cytoskeleton

**Fig. 1.**
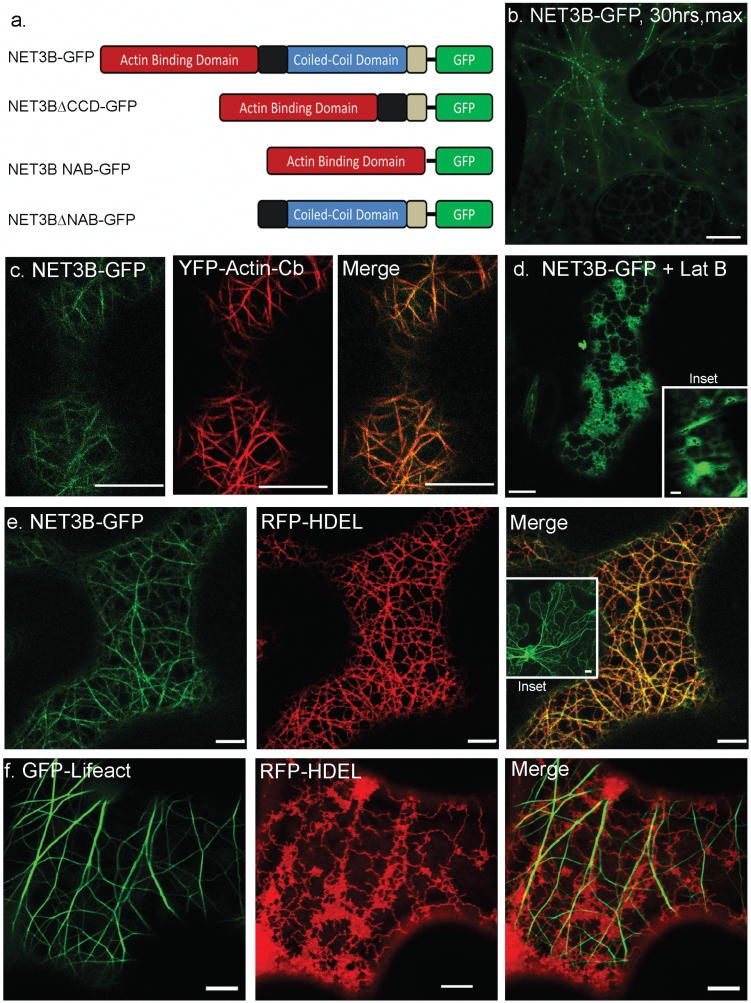
NET3B-GFP co-localizes with both the actin cytoskeleton and the ER in transformed *N. benthamiana* leaf epidermal cells. *N. benthamiana* leaf epidermal cells transiently transfected with fluorescent protein constructs either singly or in combination with other construct(s) as shown in each panel. (a) Graphical illustration of the construction of NET3B fusions. (b) 3D maximum projection of NET3B-GFP expressing cells with low expression. NET3B-GFP labels numerous actin cytoskeleton-associated puncta, producing the typical ‘beads-on-a-string’ pattern characteristic of NET family proteins. (c) NET3B-GFP/YFP-actin-Cb expressing cells. NET3B-GFP localizes to filamentous structures that are also labelled with the actin marker. (d) NET3B-GFP redistributes to the ER membrane when the actin cytoskeleton is depolymerised by latrunculin B. The inset picture shows that GFP-Lifeact becomes cytoplasmic after latrunculin B treatment. (e) NET3B-GFP/RFP-HDEL expressing cells. The expression of NET3B-GFP enhances the association between the ER and the actin cytoskeleton. Consequently, the morphology of the RFP-HDEL labelled ER network shows greater alignment with the actin network. The inset picture shows that full length NET3B without a tag is also able to enhance the association between the actin cytoskeleton and the ER. (f) GFP-Lifeact/RFP-HDEL expressing cells. The actin and ER networks exhibit little alignment with each other in contrast to (e). Lat B, latrunculin B. Scale bar, 10 µm.

Arabidopsis NET3B cDNA was fused in frame with GFP at its C-terminus (Fig. 1a) and transiently expressed in *N. benthamiana* leaves using the infiltration method (Sparkes *et al.*, 2006). At low expression levels, achieved up to 30 hours after infiltration, NET3B-GFP labelled F-actin associated punctae (Fig. 1b), producing a ‘beads-on-a-string’ pattern that is characteristic of members of the NET family (Deeks *et al.*, 2012). At high expression levels, achieved after 48 h after infiltration, NET3B-GFP labelled the entire filamentous network, which was also labelled with YFP-actin-Cb (Rocchetti *et al.*, 2014) (Fig. 1c). This indicates that NET3B, like other members of the NET family, associates with the actin cytoskeleton *in vivo*.

When the actin cytoskeleton was labelled using the marker Lifeact, subsequent treatment with the actin depolymerising drug latrunculin B caused the marker to become cytosolic (Fig. 1d, inset). In contrast, NET3B-GFP localized to the ER upon treatment with latrunculin B, suggesting that localizeNET3B-GFP has the ability to bind to the ER (Fig. 1d). Interestingly, ER morphology is significantly altered by high expression of NET3B-GFP. Under normal conditions, when GFP-Lifeact was used as a marker in leaf cells, the labelled actin cables and ER network appeared intertwined with each other (Fig. 1f) but with little co-localization. However when overexpressing NET3B-GFP, the ER network and actin cytoskeleton showed clear co-localization (Fig. 1e). Under normal conditions, the ER network comprises membrane cisternae interlinked with tubular structures. However few ER cisternae were observed when NET3B-GFP was overexpressed and some parts of the ER network appeared to follow the pattern and organization of the NET3B-GFP-labelled actin cytoskeleton (Fig. 1e). When actin was depolymerised in the presence of NET3B-GFP, the morphology of the ER network was restored, suggesting it was no longer constrained by any potential NET3B-actin interaction (Fig. 1d). Expressing full length NET3B without a fluorescent protein tag produced a similar effect on the ER as NET3B-GFP, indicating that the GFP tag does not significantly affect its activity (Fig. 1e, inset).

**Fig. 2.**
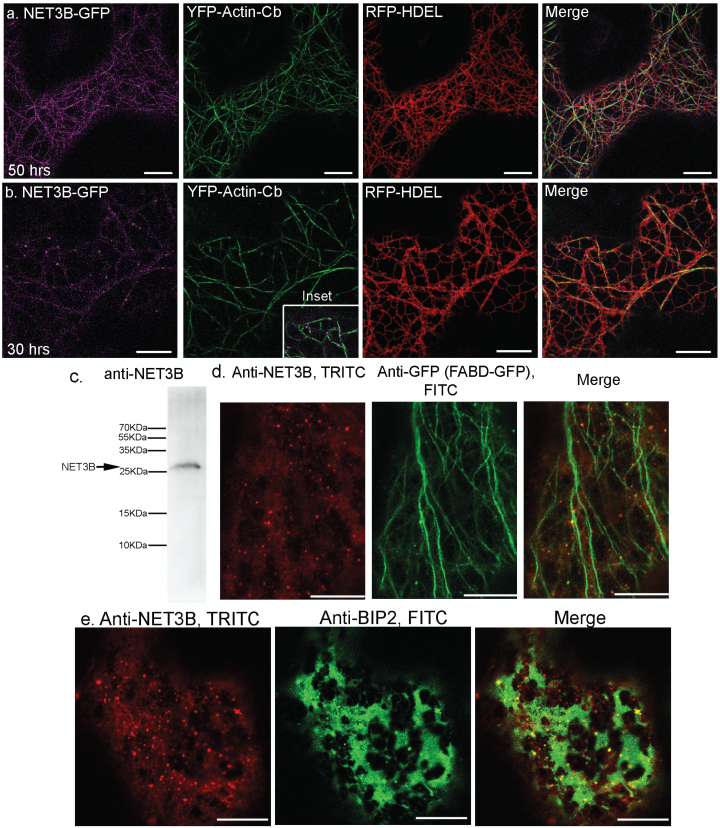
Endogenous Arabidopsis NET3B co-localize with the actin and ER networks. (a–b) NET3B-GFP (magenta) co-expressed with YFP-actin-Cb (green) and RFP-HDEL (red) at different time points after infiltration into *N. benthamiana* leaves. The same detection settings were used for imaging GFP. Strong alignment between the ER and actin cytoskeleton was seen in (a) but not in (b). The NET3B puncta seen in (b) align with the actin cytoskeleton (inset) and are associated with the ER membrane. (c) Western blot of total protein extract from Arabidopsis seedlings probed with a polyclonal NET3B antibody (anti-NET3B) showing a clear band at around 25 kDa. (d) Immunofluorescence images of cotyledon cells of an Arabidopsis line stably expressing FABD2-GFP, which marks actin. Cells stained with anti-NET3B (TRITC, red) and anti-GFP antibodies (FITC, green). Endogenous NET3B was detected in both the cytoplasm and as punctate structures, which aligned with the actin network. (e) Immunofluorescence images showing that the anti-NET3B labelled puncta also associated with the ER network, which is labelled with anti-BIP2 (FITC, green). Scale bar, 10 µm.

The effect of NET3B on ER morphology at different expression levels is better illustrated by the triple expression of NET3B-GFP, YFP-actin-Cb and RFP-HDEL in cells (Fig. 2a, b). Note the strong ER-actin alignment observed under high levels of NET3B-GFP, while under low levels of NET3B-GFP only puncta of ER are seen to align along the actin cytoskeleton (as in Fig. 1a).

We studied the localization of endogenous NET3B in Arabidopsis cotyledon cells using a NET3B polyclonal antibody raised in mice. This antiserum detects a single band on gel immunoblots of Arabidopsis protein extracts (Fig. 2c). In Arabidopsis cotyledon cells, endogenous NET3B localized to puncta, which co-localized with both the actin cytoskeleton (Fig. 2d) and the ER membrane (Fig. 2e). This result is in agreement with the localization of NET3B-GFP expressed at low levels in *N. benthamiana* leaves (Figs 1a and 2b). Taken together, these data indicate that NET3B localizes to specific ER domains that associate with the actin cytoskeleton. When NET3B-GFP expression in *N. benthamiana* leaf epidermal cells is high, it localizes throughout the ER membrane and appears to enhance the association of the ER with actin. This is probably because there is more NET3B available to mediate this interaction.

### NET3B has two functional domains

**Fig. 3.**
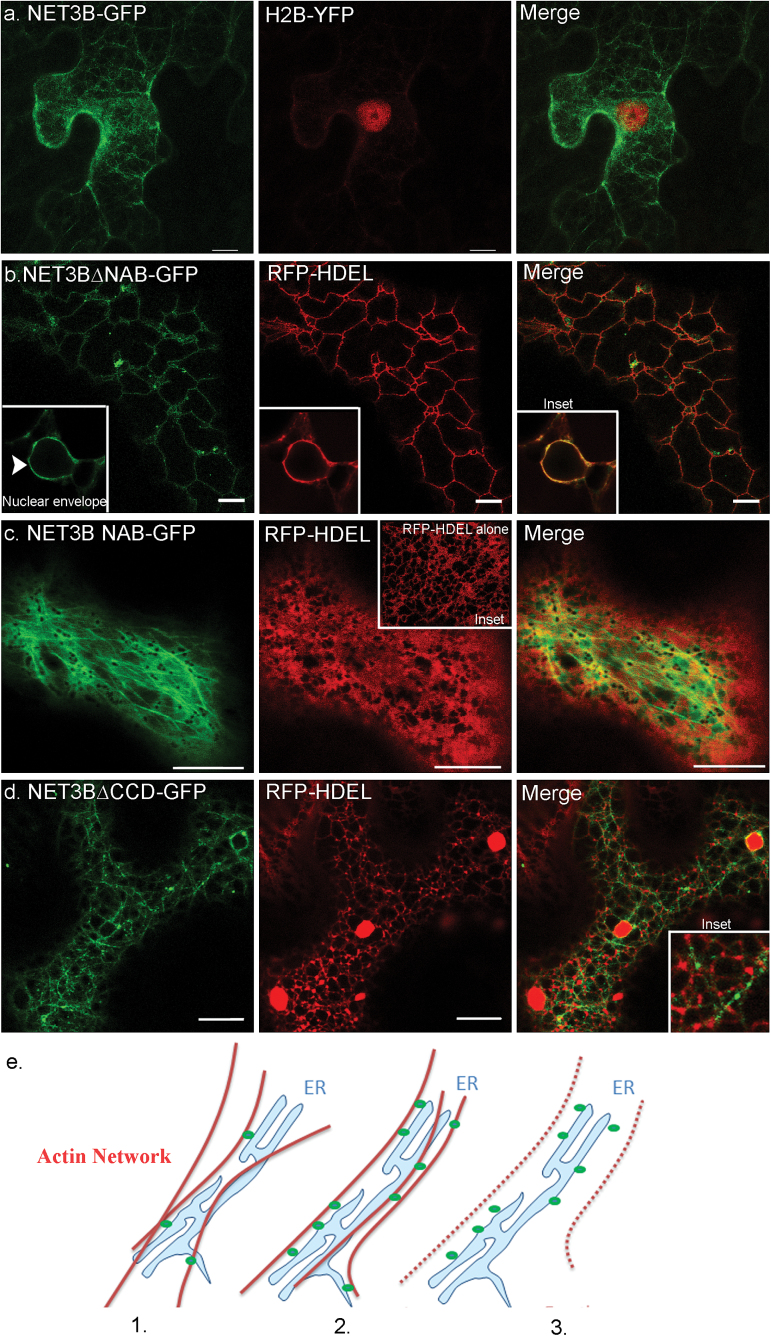
NET3B co-localizes with the actin and ER networks through its N-terminal NAB domain and a C-terminal ER binding domain, respectively. Domain deletion mutants of NET3B were fused in frame to GFP and transiently expressed with RFP-HDEL in *N. benthamiana* leaf epidermal cells. (a) 3D projection of cells expressing NET3B-GFP and the nuclear marker H2B-YFP. Note: unlike most ER membrane proteins, NET3B-RFP does not label the nuclear envelope. (b) NET3B∆NAB-GFP/RFP-HDEL expressing cells. NET3B∆NAB localizes to the ER network and the nuclear envelope (inset). (c) NET3B-NAB-GFP expressing cells. NET3B-NAB-GFP labels the actin cytoskeleton, with a strong cytoplasmic background. Under these conditions, the ER network becomes more cisternae-like when compared with cells expressing RFP-HDEL (inset). (d) NET3B∆CCD-GFP expressing cells. NET3B∆CCD-GFP labels the actin cytoskeleton, with numerous puncta aligned along the actin network. The ER network is disrupted and its association with the NET3B∆CCD-GFP labeled actin cytoskeleton is much reduced. (e) Proposed model of NET3B interaction with the ER. (1) NET3B associates with the ER and the actin cytoskeleton at specific membrane foci. (2) NET3B overexpression brings the actin cytoskeleton and the ER network closer together by increasing the number of sites of interaction. (3) NET3B localizes to the ER when the actin cytoskeleton depolymerises. Scale bar, 10 µm.

NET3B-GFP is not observed on the nuclear envelope (NE), which is continuous with the ER network. The NE harbours many ER proteins and the lack of NET3B-GFP here indicates that the membrane association of NET3B is specific (Fig. 3a). Two distinct domains are found in the NET3B sequence, namely an N-terminal NET actin binding (NAB) domain and a C-terminal coil-coiled domain. Direct association between the NAB domain of NET1 and NET4 with F-actin has been demonstrated in our previous studies (Deeks *et al.*, 2012). Unfortunately, due to the insolubility of NET3B and its NAB domain it has not been possible to verify direct binding *in vitro*. However its association with the actin cytoskeleton is supported by its localization *in vivo* (Fig. 1b, c).

**Fig. 4.**
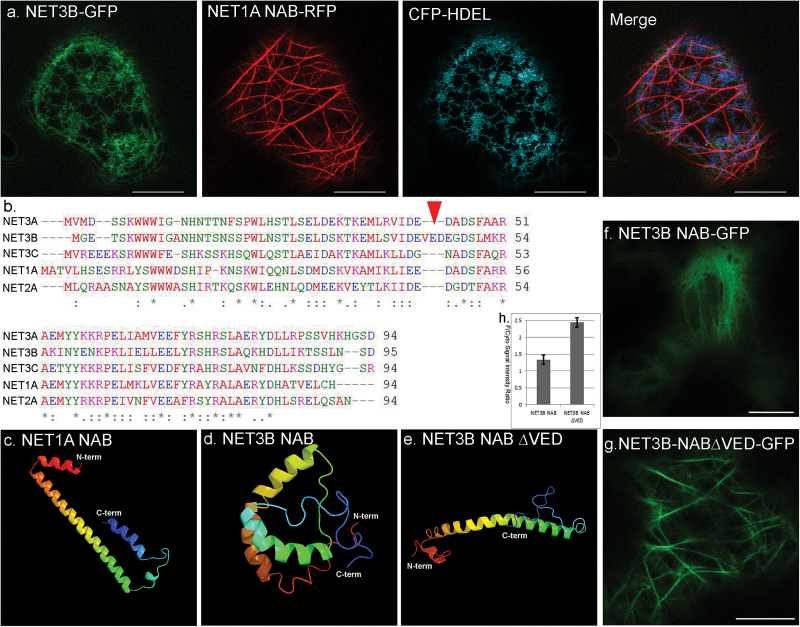
NET3B has an insertion in its NAB domain that reduces its ability to associate with the actin cytoskeleton *in vivo*. (a) *N. benthamiana* leaf epidermal cells expressing NET3B-GFP/NET1A-NAB-RFP/CFP-HDEL. NET3B-GFP is not able to associate with the actin cytoskeleton in the presence of NET1A-NAB-RFP. Note: the actin cytoskeleton is only labelled with NET1A-NAB-RFP and NET3B-GFP localizss to the ER as determined by its co-localization with CFP-HDEL. (b) Sequence alignment of the NAB domain from NET1A, NET2A, NET3A, NET3B and NET3C. The three amino acid insertion, VED, is a unique feature of NET3B. (c–d) Predicted 3D structures of the NAB domains listed in (b) analysed using Phyre2. Significant differences are seen in the 3D structure of the NAB domains of NET1A NET3B. The arrow indicates the approximate position of the VED insertion of NET3B. (e) Once the VED motif is removed from the NAB domain of NET3B, its predicted 3D structure is very similar to NET1A. (f–g) *N. benthamiana* leaf epidermal cells expressing NET3B-NAB-GFP and NET3B-NAB∆VED -GFP where the VED motif is absent. A stronger cytoplasmic signal is seen in NET3B-NAB-GFP expressing cells. (h) Quantification of the ratio between the fluorescence intensities of the cytoplasm and the actin cytoskeleton in (f–g), further illustrating the stronger cytoplasmic signal seen in NET3B-NAB-GFP expressing cells. Scale bar, 10 µm.

Domain deletion mutants of NET3B cDNA were made and fused in frame to GFP (Fig. 1a). These were used to transiently transform *N. benthamiana* leaf epidermal cells. NET3B without the NAB domain, NET3B∆NAB-GFP, localized to the ER network as well as the nuclear envelope (Fig. 3b), while the N-terminal NAB domain alone co-localized with the actin cytoskeleton (Fig. 3c). This suggests that the NET3B C-terminus, including the coil-coiled sequence, is involved in the interaction of NET3B with the ER. When NET3B-NAB-GFP was expressed in the leaf epidermal cells, strong cytoplasmic background fluorescence was observed, suggesting that its localization to the actin cytoskeleton is not as efficient as the full length NET3B-GFP (discussed later in Fig. 4). This phenomenon is not observed when using the NAB domains of other NET proteins in similar experiments (Deeks *et al.*, 2012). This has two potential implicatons: firstly, that the co-localization of the NAB domain of NET3B with the actin cytoskeleton is lower than that of other NET proteins and secondly, that the ER association of NET3B is important for its localization to the actin cytoskeleton. ER cisternae were also formed in the presence of NET3B-NAB-GFP, with fewer ER tubular structures observed compared with that of wild type cells (Fig. 3c, inset).

NET3B without the coiled-coil domain, NET3B∆CCD-GFP, localizes to numerous puncta that align with the actin cytoskeleton (Fig. 3d). However, this deletion mutant is unable to interact with the ER membrane as little co-localization between the ER and the chimeric protein is seen (Fig. 3c, inset). Moreover, the morphology of the ER network is disrupted, with more ER aggregates in the presence of NET3B∆CCD-GFP. This suggests that the coiled-coil domain deletion mutant has a dominant negative effect, whereby it interferes with the normal function of the endogenous protein. Alterations in ER morphology were observed when these NET3B deletion mutants were expressed. This could be because the interaction between NET3B and other ER structural proteins is disturbed.

Bearing these data in mind, we propose a model for the association of NET3B with the ER and the actin cytoskeleton. In this model NET3B behaves as a linker protein; it associates with the actin cytoskeleton through the N-terminal NAB domain, and to the ER membrane through its C-terminus, which requires the coiled-coil domain. This is supported by the following evidence: (1) *In planta* immunolocalization studies in Arabidopsis have shown that NET3B localizes to specific ER domains, where it is also able to associate with actin; (2) when overexpressed in *N. benthamiana* NET3B-GFP appears to distribute across the whole actin and ER network, resulting in enhanced association between the ER and actin; (3) when actin is disrupted NET3B-GFP localizes to the ER (Fig. 3e).

### NET3B has a unique NAB domain

When full length NET3B-GFP is co-expressed with the NAB domain from NET1A, NET1A-NAB-RFP, in *N. benthamiana* epidermal cells, NET3B-GFP distributed to the ER surface and to the cytoplasm but no labelling of the actin cytoskeleton was observed (Fig. 4a). This phenomenon is very similar to the effect of latrunculin B treatment on NET3B-GFP expressing cells (Fig. 1d). These data suggest that the NAB domain of NET1A, which is known to directly bind to F-actin, interferes with the association of NET3B with the actin cytoskeleton. The NAB domain of NET1A and NET3B are therefore likely to interact with the actin cytoskeleton at the same site, with the NAB domain of NET1A having a higher affinity for this site.

The conserved N-terminal NAB domain is a characteristic feature of the NET superfamily of proteins. However the NAB domain of NET3B is unique in having an insertion of three amino acids, namely valine, glutamic acid and aspartic acid (VED) (Fig. 4b). The 3D structures of the NAB domain in NET1A, NET3B and NET3B∆VED were analysed *in silico* using Phyre2. Interestingly, the predicted structure of the NAB domain of NET3B is significantly different from the NAB domain of NET1A. This is because the VED insertion induces a beta turn (Fig. 4c, d). However, by removing this insertion, as in NET3B∆VED, the structure of the NAB domain of NET3B reverted to a structure similar to the NAB domain of NET1A (Fig. 4e). With this in mind, the NAB domain of the NET3B construct was manipulated to remove the VED motif. This construct, NET3B-NAB∆VED-GFP, was expressed in parallel with NET3B-NAB-GFP under the same conditions. The cytoplasmic background of NET3B-NAB-GFP is much stronger than of NET3B-NAB∆VED-GFP, as illustrated by the signal intensity ratio of actin filaments relative to the cytoplasm (Fig. 4f–h). These data indicate that the ability of NET3B to associate with actin is enhanced by removing the VED motif from its NAB domain. The NET3B∆VED-GFP mutant protein is still able to link the actin and ER networks, which is as expected given that the C-terminus of NET3B is unaffected and is responsible for its ER localization (Supplementary Fig. S1a, b).

### NET3B-GFP overexpression restricts membrane diffusion

**Fig. 5.**
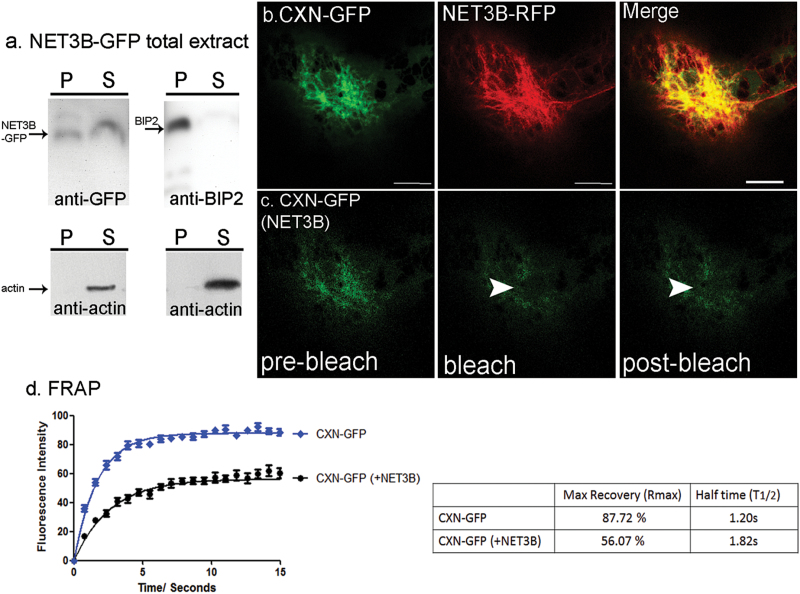
NET3B-GFP overexpression restricts membrane diffusion. (a) Western blots of high-speed ultracentrifuged pellets and supernatants from *N. benthamiana* leaf extracts expressing NET3B-GFP. (i) Anti-GFP antibody shows that NET3B-GFP is detected in the pellet, which harbours the ER membrane microsomal fraction and in the supernatant. Anti-actin antibody shows that actin is only present in the supernatant; (ii) The ER protein BIP2 is found only in the pellet, as detected by anti-BIP2 antibody. Again, anti-actin antibody revels actin is only present in the supernatant. Note: NET3B-GFP has the characteristics of a peripheral membrane protein. (b-c) Representative images of photobleached ER membrane imaged for CXN-GFP in the presence of NET3B-RFP. (d) FRAP analysis of CXN-GFP in CXN-GFP/NET3B-RFP expressing cells compared with cells only expressing CXN-GFP. Note: Movement of the ER membrane, as depicted by the recovery of CXN-GFP fluorescence, is signifcantly retarded in the presence of NET3B-RFP. This is reflected by a prolonged T_1/2_ and reduced Rmax. Scale bar,10 µm.

We used high-speed centrifugation to determine whether NET3B is a peripheral membrane protein. The data showed that after centrifugation NET3B-GFP is found in the microsomal fraction and also in the supernatant (Fig. 5a). BIP2 is a protein localized to the ER lumen. It was used as a control as it should only ever appear in the microsomal fraction, namely the pellet (Fig. 5a). Endogenous actin is only found in the supernatant as it is not able to sediment at this speed. The presence of NET3B-GFP in both the pellet and supernatant suggests that NET3B is an ER peripheral membrane protein. Its association with the ER is probably through either its interaction with other ER membrane proteins or directly with membrane lipids.

High expression levels of NET3B-GFP changes the structure of the ER by reducing the amount of membrane cisternae (Fig. 1e). In order to determine whether the expression of NET3B-RFP affected membrane diffusion we performed fluorescence recovery after photobleaching (FRAP) on the ER protein calnexin (CXN), in the presence or absence of NET3B-RFP. In the presence of NET3B-RFP, the recovery of CXN-GFP in the photobleached region was much slower than in the absence of NET3B-RFP and a large immobile fraction was also observed (Fig. 5b–d). These data indicate that the interaction of NET3B with the ER can affect ER membrane diffusion.

### Characterization of NET3B T-DNA mutants

**Fig. 6.**
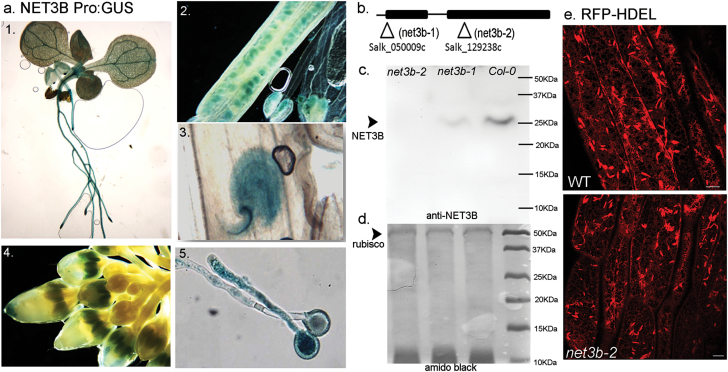
NET3B mutants, *net3b-1* and *net3b-2*, exhibit no significant effects on the ER network. (a) NET3B::GUS expression in stably transformed Arabidopsis lines. GUS staining was seen in seedlings (1), developing seeds (2–3), anther/pollen (4) and pollen tubes (5). (b) Illustration of the position of the T-DNA insertions in *net3b-1* and *net3b-2* lines. (c) Western blot of total protein extracts from Arabidopsis flowers of Col-0, *net3b-1* and *net3b-2*, probed with anti-NET3B antibody. Note: NET3B is absent from *net3b-2*, which a knockout mutant, while *net3b-1* shows significantly reduced NET3B expression. (d) The amido black stain indicates equal protein loading. (e) Both wild type and *net3b-2* plants are transformed with RFP-HDEL. Hypocotyl epidermal cells are shown. Note: the ER organisation looks similar and no obvious defects can be observed. Scale bar, 10 µm.

The expression pattern of NET3B was studied using the GUS reporter gene system. The NET3B promoter, 2 kb upstream of the translation start codon, was fused in frame with the GUS reporter cDNA and the construct used to transform Arabidopsis. GUS was expressed in various tissues including pollen, embryos, roots and the leaf vasculature (Fig. 6a). A high level of NET3B promoter activity was also observed in the meristematic cells of the root tip.

We identified two homozygous NET3B T-DNA mutant lines: in *net3b-1* the T-DNA insertion is in the promoter and in *net3b-2* it is in the second exon (Fig. 6b). Through western blotting with anti-NET3B antibody, we found that NET3B protein levels are reduced in *net3b-1* and absent in *net3b-2.* This result further confirmed the specificity of the NET3B antibody as no other protein bands were detected in the knockout mutant *net3b-2* (Fig. 6c, d). Both mutants exhibit normal growth and development and have no significant morphological defects when compared with wild type. The ER organization in the *net3b-2* mutant was further analyzed by stably expressing RFP-HDEL, which is retained in the ER. In regards to ER morphology, no significant phenotypes were observed (Fig. 6e). It is known that multiple proteins are required for actin-myosin based ER movement and that the overall structure of the ER network is not affected significantly when the actin cytoskeleton is removed chemically. Thus depleting one protein i.e. NET3B may not be sufficient to produce significant defects in ER morphology.

In conclusion, we have shown that NET3B is a novel membrane adaptor protein, which links membranes to the actin cytoskeleton. NET3B associates directly with the ER and co-localizes with the actin cytoskeleton. NET3B may also be involved in dictating ER morphology and dynamics as overexpression affects both the structure of the ER and diffusion within the ER membrane. Furthermore, the NAB domain of NET3B contains a unique three amino acid insertion (VED), which reduces its ability to associate with the actin cytoskeleton *in vivo* when compared with NET1A, which has been shown to bind F-actin directly. How NET3B can modulate ER structure remains to be determined but it is possible that NET3B works in conjunction with other ER localized actin regulatory or motor proteins. For example, the dynamics of the ER network in plants is modulated by the myosin XI family, which contain a motor domain used for driving actin-based movement. It is possible that NET3B provides an anchorage mechanism that facilitates the process of myosin-based ER movement. Taken together our study suggests that NET3B is a novel protein involved in actin cytoskeleton based ER modelling.

## Supplementary Material

Supplementary Data

## Abbreviations:

CXN: calnexin
EPCS: endoplasmic reticulum-plasma membrane contact sites
ER: endoplasmic reticulum
GFP: green fluorescent protein
NAB: NET actin binding
NE: nuclear envelope
NET3B: networked 3B
