# Supplementary material for: NETWORKED 3B: a novel protein in the actin cytoskeleton-endoplasmic reticulum interaction
Source: J Exp Bot. 2017 Mar 28;68(7):1441–50. doi: 10.1093/jxb/erx047 (PMC5441911; doi:10.1093/jxb/erx047)

NETWORKED3B (NET3B): a novel protein involved in the actin cytoskeleton-endoplasmic reticulum interaction.

Pengwei Wang and Patrick J Hussey

Supplementary Table 1. List of Primers used in this study.

Name of Primer	Sequence (5'-3')
NET3B-F	gggg aca agt ttg tac aaa aaa gca ggc ttc ccg cca ATG GGT GAG ACA TCA AAA TGG TG
NET3B-R	gggg acc ac ttt gta caa gaa agc tgg gtc AAA CGA AAA CAT TAT GAG AAA ATA GTA AC
NET3B NAB-R	gggg aca agt ttg tac aaa aaa gca ggc ttc ccg cca GAT CAA GAG ATC ATG CTT CTG
NET3B NAB-F	Same to NET3B-F
NET3BΔCCD-F	CAA ATT GTT GAA TTT GAC GAT GGT GTT TGC TTT TGC TTC CAA TTC A
NET3BΔCCD-R	TGA ATT GGA AGC AAA AGC AAA C ACC ATC GTC AAA TTC AAC AAT TTG
NET3BΔNAB-F	gggg aca agt ttg tac aaa aaa gca ggc ttc ccg cca ATG AAAACCTCT TCTCTGAATTCGG
NET3BΔNAB-R	Same to NET3B-R
NET3B ab-F	gggg aca agt ttg tac aaa aaa gca ggc ttc ccg cca ATG GTG ACT CGG TTG CTT GCA A
NET3B ab-R	Same to NET3B-R
NET3BΔVED-F	CTCATCAATCACGCTCAGC GAAGGAGATTCCTTGATGAAAC
NET3BΔVED-R	GTTTCATCAAGGAATCTCCTTC CTCATCAATCACGCTCAGC
NET3B Promoter-F	gggg aca agt ttg tac aaa aaa gca ggc ttc ccg cca ATA TCT CAA TTA TGA ATT AAT TCA ATA TAT
NET3B Promoter-R	gggg acc ac ttt gta caa gaa agc tgg gtc CGT CTT CAC TTG TAA TTT TGC AC

Supplementary Table 2. List of constructs generated in this study.

Name of Construct	Expression Vector	Cloning Primers
NET3B-GFP	pMDC83-GFP	NET3B-F + NET3B-R
NET3B-RFP	pMDC83-RFP	NET3B-F + NET3B-R
NET3B NAB-GFP	pMDC83-GFP	NET3B-NAB-F + NET3B-NAB-R
NET3BΔNAB-GFP	pMDC83-GFP	NET3BΔNAB-F + NET3B-R
NET3BΔCCD-GFP	pMDC83-GFP	NET3BΔCCD-F + NET3BΔCCD-R
NET3BΔVED-GFP	pMDC83-GFP	NET3BΔVED-F + NET3BΔVED-R
NET3B-GUS	pBI101-GUS	NET3B Promoter-F + NET3B Promoter-R
NET3B antigen	pGAT4-His	NET3B ab-F + NET3B ab-R

Figure S1. NET3B has an insertion in the NAB domain that reduces its actin associating ability but not its ER binding ability.

(a) NET3B without the VED motif is fused in frame to GFP. In NET3BΔVED-GFP/ NET1A NAB-RFP co-expressing cells, interestingly, actin cytoskeleton co-labelling with both proteins are observed. **(b)** NET3BΔVED-GFP and RFP-HDEL coexpressing cells. NET3BΔVED is still able to associate with the ER membrane (scale bar = 10μm).

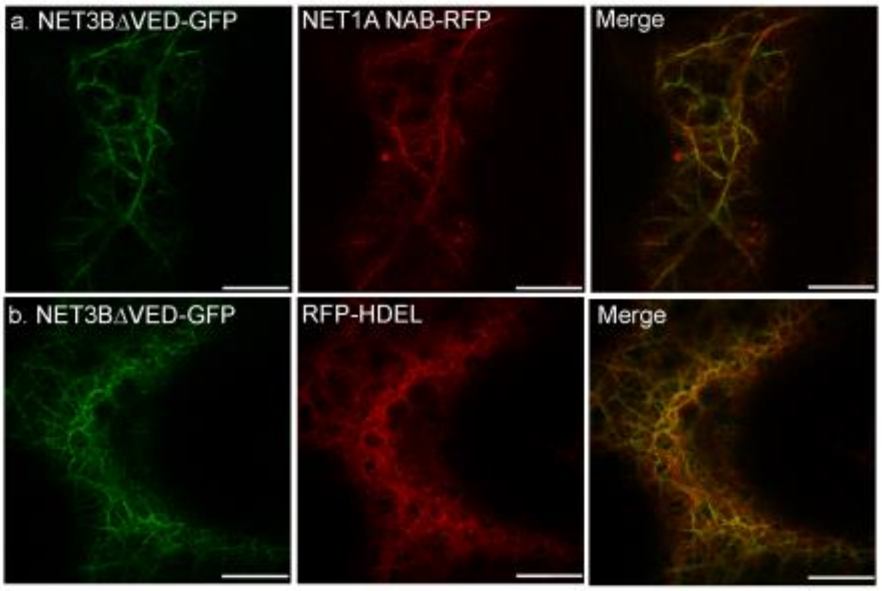

Supplement: Supplementary Data [file erx047_Supplementary_Data.zip › supplementary_table_S1_S2_figure_S1.pdf]
